# Pediatric breast lymphatic malformation with recurrent presentation in an adolescent female

**DOI:** 10.1259/bjrcr.20210077

**Published:** 2021-09-10

**Authors:** Rachel Kaye, Rebecca Leddy

**Affiliations:** 1 Medical University of South Carolina, Charleston, South Carolina, United States; 2 Department of Radiology and Radiological Science, Medical University of South Carolina, Breast Imaging, Charleston, South Carolina, United States

## Abstract

This case report summarizes a rare case of left chest wall/breast lymphatic malformation or cystic lymphangioma in a female child of 18 months with multiple late recurrences in adolescence. By maternal history, the mass was excised initially, but the patient presented at age 15 and 17 years for recurrences and associated symptoms. Comments focus on a complex clinical history and treatment management of patient symptoms and concerns. Breast sparing treatments were employed with sclerotherapy and the T lymphocyte inhibitor, Sirolimus (Rapamune).

## Introduction

Lymphatic malformations are uncommon benign vascular malformations which can occur anywhere in the body.^1^ They can be classified as congenital or acquired. Congenital lymphatic malformations are thought to arise from embryologic sequestration of lymphatic tissue with failure of lymphatic channels to connect to the remainder of the lymphatic/venous system.^2^ Almost all (95%) congenital malformations of the lymphatic system occur in the head-neck region and in the axilla and are diagnosed typically by the age of two years.^3^ Lymphatic malformation of the breast is rare, most often affecting young adult females, and it is especially uncommon among female infants.^4^ The same investigators found only 17 cases of lymphatic malformation of the breast described in the literature, and of these, four cases were among children, and one occurred in a pediatric female patient.^4,5^ Thus, it is rare to see a pediatric-aged female patient with lymphatic malformation of the breast. Gupta et al.^4^ did document a case of lymphatic malformation of the breast in an 8-year-old male child who was treated by excision.^4^ In 2004, Al-Salem reported 22 children (12 female and 10 male) treated for lymphatic malformation.^6^ Only one child had breast lymphatic malformation. This child was a one-year-old girl who presented with left breast enlargement and was treated by surgical excision. Histology proved the mass to be lymphangioma involving the breast. In 2014, Minocha et al. documented an eight-year-old girl with lymphatic malformation of the breast and a left axillary cystic mass that was surgically excised.^7^ These previously described cases in the literature demonstrate evidence of similar initial breast presentations as the patient described in this case; however, this presented case report provides follow-up of a complex clinical course over an extended period.

According to Gupta et. al. 2011,^4^ ultrasound studies of lymphatic malformation of the breast tend to reveal a multiloculated, hypoechoic, cystic mass, with linear septa of variable thickness that contain solid elements originating from the cyst walls or septa.^4^ Yet, magnetic resonance imaging (MRI) is the modality of choice for diagnosis and evaluation of a lymphatic malformation of the breast.^4^ On MRI, lymphatic malformation is typically seen as a septated mass of low T1- and high *T*_2_-weighted signal intensity, with variable enhancement from septae only. Since lymphatic malformation may be related to genetic syndromes in pediatric patients, genetic studies of possible lymphatic malformation syndromes, such as Noonan and PIK3CA syndromes, were conducted. Associated anomalies of lymphatic malformations are Turner’s syndrome, Down’s syndrome, Trisomy 18, Trisomy 13, and Noonan syndrome.^8^

## Case report

A 15-year-old female adolescent was referred by her pediatrician to pediatric surgery for a left lower quadrant breast mass. By maternal report, the patient showed a mass in the same location when she was an infant of 18 months, and it was resected and diagnosed as lymphatic malformation. The patient’s mother stated that subsequently the girl experienced mass recurrences in the same region, often appearing to coincide with times of illness or stress. It was reported that typically these recurrences spontaneously resolve, are not painful, and do not exceed the size of a quarter. However, the mother characterized the present mass at the inferior lateral aspect of the left breast as progressively enlarged over the past few weeks, painful in certain body positions, irritated by her inferior bra line, and not resolving as in the past. The patient denied changes to the mass related to her menstrual cycle, and she denied recent illnesses, trauma, or major changes in activity or lifestyle. She endorsed a one-to-two-month history of increased fatigue and achy joints. Past surgical history was remarkable for tonsillectomy at age four with enlargement secondary to lymphatic congestion. No family history was contributory.

**Figure 1.**
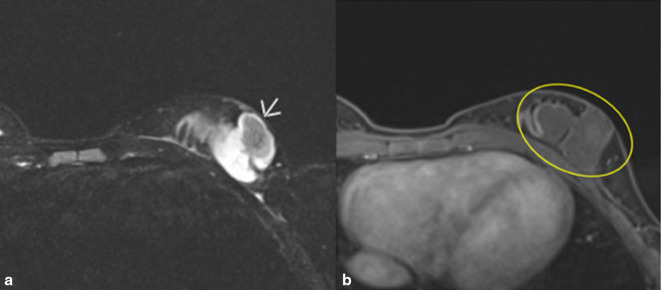
A and B. MRI images of left breast with contrast. (**A**). *T_2_*-weighted axial fat-suppressed sequence. Arrow shows a lobulated cystic multioculated mass in the left lower outer breast measuring 4.9× 2.7 × 5.6 cm superior to the patient’s scar consistent with lymphatic malformation. (**B**). T1 axial postcontrast and fat-suppressed sequence. No significant internal enhancement. Minimal rim enhancement. Mass goes to skin and close proximity to chest wall without invasion.

Physical exam revealed tenderness of the left breast and a 3–4 cm poorly circumscribed spongy mass extending from the left lower quadrant of the breast superiorly to the areola. A well-healed 2-cm linear incision scar was noted inferior to the mass. Overlying skin features were consistent with vascular abnormality. There were mild nipple changes, including mild retraction but incomplete inversion. There was left axillary lymphadenopathy and left axillary pectoral adenopathy. MRI images of both breasts with and without contrast showed a 4.9 × 2.7 × 5.6 cm lobulated multiloculated mass with minimal rim enhancement and no malignant features, consistent with recurrent lymphatic malformation (Figure 1).

**Figure 2.**
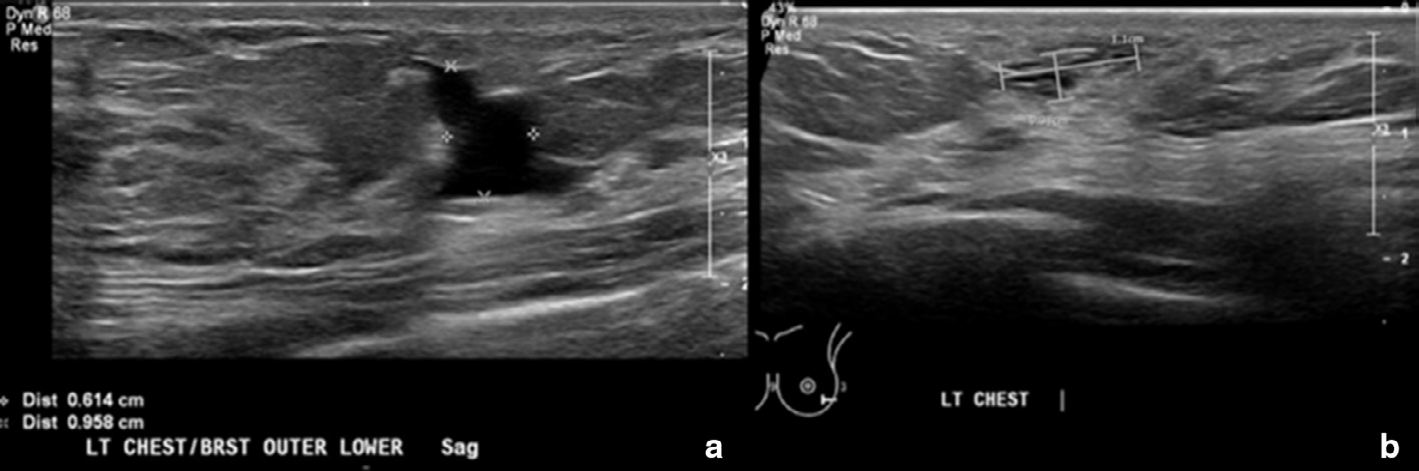
A and B. Ultrasound images of the left breast from 6-month follow-up after sclerotherapy and a two-year follow-up. (**A**). Image shows the left breast/chest wall lymphatic malformation status postsclerotherapy treatment. There is an irregularly shaped anechoic lesion in the left lower outer breast measuring 1.3 × 0.6 × 1.0 cm. No internal flow with color Doppler. (**B**). Image shows the left breast/chest wall lymphatic malformation after a two-year follow-up. There are small, irregularly shaped anechoic lesions within the outer lower left breast and within the chest wall inferior to the left breast. The portion within the left breast measures 1.1 × 0.4 cm. There is a decrease in size of the left breast/chest wall lymphatic malformation compared to 1.3 × 0.6 cm previously. There is no macrocystic component as well as seen on the prior ultrasound. No internal vascular flow is noted by color Doppler.

Treatment was initiated after consultation with plastic surgery, interventional radiology, and the lymphatic malformation specialty group. Consensus recommendation was for sclerotherapy as opposed to operative intervention for the initial phase of left chest wall/breast lymphatic malformation treatment. The patient and her mother consented to the procedure. At 15 years of age, the patient underwent one interventional radiology fluoroscopically guided sclerotherapy treatment of the left breast lymphatic malformation. Utilizing a 21 G angiocath needle, the left breast lymphatic malformation was directly punctured under ultrasound guidance. Approximately 26 cc of sanguineous fluid were aspirated. Then, 20 ml of doxycycline mixed with contrast (10 mg ml^−1^) was injected under fluoroscopy into the malformation, which demonstrated slow accumulation of contrast with no systemic venous drainage. The procedure was performed under general anesthesia with no complications. She presented for follow-up one month after the procedure. On this visit, she reported no complaints, significant pain improvement, and decreased size of the breast mass. Physical exam affirmed decreased size of the lymphatic malformation on the left chest wall/breast. No ulcers or bleeding were present. Follow-up was scheduled every six months. Six months later, the patient presented to pediatric surgery and reported that the area of her lymphatic malformation felt full at times. Ultrasound revealed an irregularly shaped anechoic lesion in the left lower outer breast measuring 1.3 × 0.6 × 1.0 cm, previously 1.9 cm in the largest diameter. No internal flow with color Doppler was present (Figure 2A).

A repeated ultrasound of the breast was performed at a two-year follow-up and revealed a decrease in size (1.1 × 0.4 cm) and no blood flow of the lesion (Figure 2B). Despite her recovery, the patient reported unusual symptoms and was diagnosed with pectus carinatum, which was deemed mild and treated with a brace. She was referred for genetic studies for possible lymphatic malformation syndromes, such as Noonan and PIK3CA syndromes. No pathogenic mutations were found on Noonan and Comprehensive RASopathy panel with 90% confidence.

**Figure 3.**
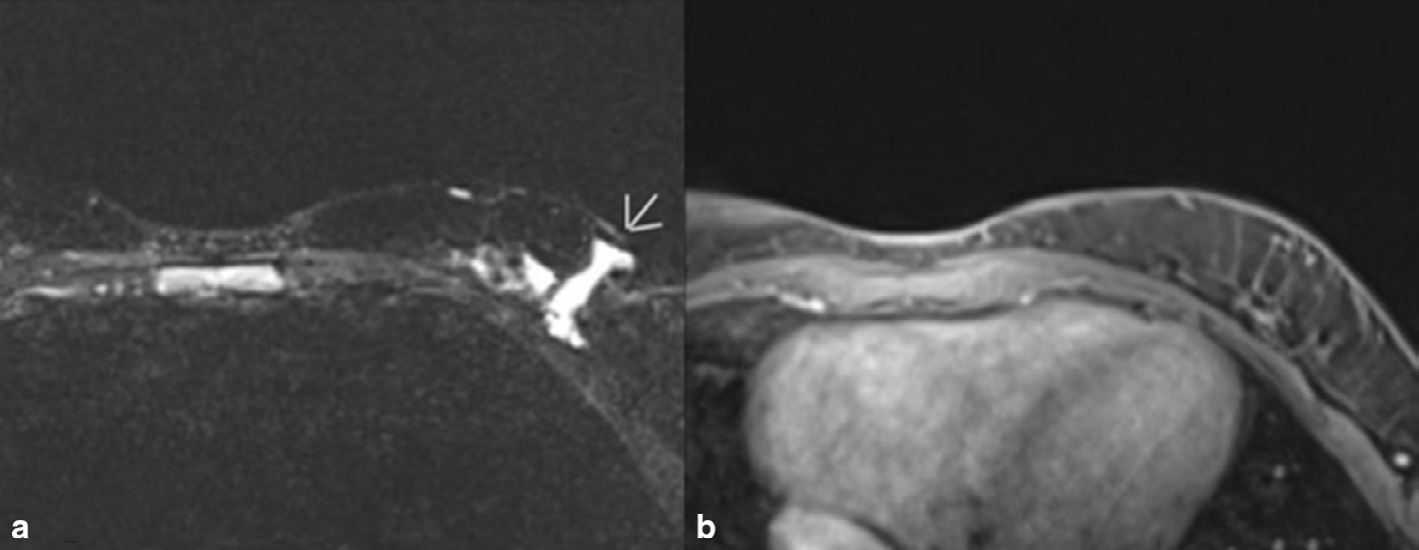
A and B. MRI images of left breast with contrast most recently. (**A**). Image shows recurrence of lymphatic malformation. Impression was overall benign with 2.6 × 0.5 cm serpinginous area of T2 hyperintense signal in the lower outer quadrant of the left breast with hyperintense T2 signal extending inferolaterally with the distal aspect excluded by collimation. These findings are located at the surgical bed and are suspicious for residual disease. (**B**). *T_1_* weighted fat-suppressed contrast-enhanced sequence no significant enhancement.

Two years later, the patient presented again with concern that the breast mass was recurring. She showed intermittent swelling of the left axilla which now involved the left postscapular area. An MRI of the breast bilaterally with and without contrast showed a 2.6 × 0.5 cm serpiginous area of T2 hyperintense signal in the lower outer quadrant of the left breast with hyperintense T2 signal extending inferior laterally. These findings were located at the surgical bed and were suspicious for residual disease (Figure 3). The treatment plan specified management of the lymphatic malformation with sirolimus (Rapamune) 2 mg po BID in addition to Bactrim pcp prophylaxis. After 3 months of treatment, the patient did not see a significant change in her breast lesion while on sirolimus; however, she had a number of side effects attributed to the drug, including being tired, prolonged, and irregular menses, and increased urination and it was discontinued.

## Discussion

This case report illustrates the clinical and historical progression of a rare breast lymphatic malformation first identified in an 18-month-old female child. Treatment at the time involved surgical excision; however, maternal history raised concerns about subsequent occasional swellings at the site and discomfort over time until the child presented at age 15 with notable recurrence and related symptoms. She underwent left breast lymphatic malformation sclerotherapy with doxycycline and saw improvements at one month and later follow-ups. The patient reported significant pain reductions and size modifications. Physical examination revealed decreased size of the lymphatic malformation on the left chest wall/breast. No ulcers or bleeding were found. Nevertheless, recurrence of lymphatic malformation at the tumor site was documented within another two years. Such a history confirms clinical judgment that lymphatic malformation, although not malignant and easily visualized on radiographic studies, present notable challenges for treatment specialists whose goal is to excise or remove the malformation without recurrence. The complexities of treatment for these cases are myriad and lead to the need for long-term follow-up. Gupta et al.^4^ has observed that lymphatic malformation of the breast are locally aggressive and tend to infiltrate the surrounding tissues.^4^ As in this case, complete excision may not be possible, and lesions may recur rapidly or over time. Further, there seem to be instances of associated symptoms and physical distresses reported by patients, such as in this case, and intermittent swelling of the left axilla. Mental stress was reported by this patient, as she exhibited signs of depression. MRI changes located at the surgical bed were also suspicious for residual disease. Ongoing treatment planning at 17 years included managing the lymphatic malformation with the T lymphocyte inhibitor macrolide compound sirolimus (Rapamune) in addition to prophylactic Bactrim.

## Conclusion

The present case report focuses on a rare presentation of breast lymphatic malformation in a female infant of 18 months treated by surgical excision. Multiple recurrences over time complicated the clinical picture, and the patient required sclerotherapy at age 15 and additional treatments at 17 years. Genetic studies were performed, although no findings were of significance. The patient suffered recurring pain, swelling, and other somatic discomforts as well as depression, despite what appeared to be initial treatment benefits. Ongoing clinical and research work is needed to improve excision techniques and to offer more effective agents to enhance sclerotherapy. Breast lymphatic malformations are markedly troublesome to patients and incredibly challenging to the multidisciplinary physicians attempting to remove them completely.

## Learning points

1. It is rare to see a pediatric-aged female patient with lymphatic malformation of the breast, only 17 cases of lymphatic malformation of the breast have been described in the literature, and of these, four cases were among children, and one occurred in a pediatric female patient.
2. Lymphatic malformation, although not malignant and easily visualized on radiographic studies, present notable challenges for treatment specialists whose goal is to excise or remove the malformation without recurrence.
3. Ongoing clinical and research work is needed to improve excision techniques and to offer more effective agents to enhance sclerotherapy.
